# Effects of Drying Methods and Ash Contents on Heat-Induced Gelation of Porcine Plasma Protein Powder

**DOI:** 10.3390/foods8040140

**Published:** 2019-04-25

**Authors:** Chengli Hou, Wenting Wang, Xuan Song, Liguo Wu, Dequan Zhang

**Affiliations:** Institute of Food Science and Technology, Chinese Academy of Agricultural Sciences/Key Laboratory of Agro-Products Processing, Ministry of Agriculture and Rural Affairs, Beijing 100193, China; houchengli@163.com (C.H.); wangwentingbiome@163.com (W.W.); songxuan120@163.com (X.S.); liguowu911@163.com (L.W.)

**Keywords:** blood plasma protein powder, heat-induced gelation, drying method, ash content, texture

## Abstract

Porcine blood plasma is a rich source of proteins with high nutritional and functional properties, which can be used as a food ingredient. The plasma is usually processed into powders in applications. In the present study, the effects of drying methods and ash contents on heat-induced gelation of plasma protein powder were investigated. The drying methods had a significant impact on the gel properties of the plasma powder heat-induced gels. The hardness and elasticity of the gels by freeze-dried and spray-dried plasma powders were lower than that of the liquid plasma (*p* < 0.05). The microstructures of dehydrated plasma were denser and the holes were smaller. The secondary structure of the gels from the spray-dried plasma protein powders exhibited more α-helixes and less β-turns than that from the freeze-dried powder and liquid plasma. The thermostability of dehydrated plasma powder was found to have decreased compared to the liquid plasma. Compared with the gels obtained from the high ash content plasma protein powders, the gel from the 6% ash content plasma powder had the highest water-holding capacity and had the lowest hardness and elasticity. However, the secondary structure and microstructures of the heat-induced gels were not affected by the ash contents in the plasma powders. These findings show that the gel properties of plasma protein powder can be finely affected by drying methods and ash contents.

## 1. Introduction

Blood is one of the main coproducts from slaughtered animals. The yield of animal blood has reached approximately 10 million tons per year all around the world [1,2]. Animal blood is typically discarded as waste, which causes environmental pollution [3]. Plasma is the product of anticoagulant blood after centrifugation and the removal of blood cells. Blood plasma is a rich source of proteins with high nutritional and functional quality. Most plasma proteins are used as ingredients, mainly as binders, emulsifiers, fat replacers, polyphosphate replacements, and meat curing agents in the food industry [3,4,5,6,7]. Gel-forming ability upon heating is considered to be the most interesting attribute of plasma. Previous studies have reported that the heat-induced gelation of plasma was affected by pH and cysteine [8,9,10,11,12].

Plasma contains a complex mixture of proteins. The typical composition is 50–60% albumin, 40–50% globulins, and 1–3% fibrinogen [10]. Liquid plasma is dried to a powder for better storage and transportation. The protein powder processing eliminates many disadvantageous factors, such as perishability and difficulty to store and transport [13,14]. Spray-drying and freeze-drying are commonly used as a dehydration technique for making protein powder products. Spray-drying has many merits, such as simple operation, drying quickly, low cost, and being suitable for continuous mass production. However, spray-drying may lead to protein denaturation and conformation changes due to a higher temperature, which will probably affect the plasma protein function [15,16]. Freeze-drying can sublimate the moisture in the material directly at a low temperature by doing less harm to the structure of the protein. However, the main disadvantage of freeze-drying is the high cost. A previous study showed that the changes in temperature, moisture, and salt ions during drying could affect the quality of the protein products [17].

Ash is an important factor that can affect the quality of plasma protein powder. It mainly comes from mineral ions in blood and exogenous anticoagulants. According to previous research, the content of ash in plasma protein powder is usually up to 14% [18]. The main ingredients of ash include sodium, magnesium, and calcium, most of which is sodium [18]. Plasma protein powder with low ash content can be produced by concentrating and removing the salt with ultrafiltration and nanofiltration. Sodium chloride (NaCl) at a concentration of 1–3% is needed to facilitate protein solubilization, resulting in the gel [19]. However, the effect of ash content on the functional properties of plasma protein gel is not clear.

The objective of this study was to determine the effects of two different dehydration methods on plasma protein functionality. Additionally, on this basis, the effect of the ash content of freeze-dried plasma on the gelation properties was studied. The results can provide data support for product development and application of plasma protein powder as a food additive.

## 2. Materials and Methods

### 2.1. Materials

Fresh porcine blood was obtained from a local slaughterhouse in Beijing, China. Sodium citrate (0.345% w/v final concentration) was added to prevent coagulation. Plasma was separated by centrifugation at 2437× *g* for 8 min at 4 °C (Himac CR22 GⅡ, Hitachi, Ltd., Tokyo, Japan).

### 2.2. Preparation of Plasma Powders

Freeze-dried plasma powder was obtained after 72 h of lyophilization, under the following conditions: cold trap temperature, −60 °C; material temperature, −40 °C; vacuum, 1 Pa (LGJ-25, Four-Ring Science Instrument Plant Beijing Co., Ltd, Beijing, China). Spray-dried plasma powder (nitrogen solubility index 98.92%) was prepared using a laboratory scale spray dryer (SD-Basic, Labplant, UK) under the following conditions: inlet temperature, 150 °C; feeding speed, 0.178 mL/s. The salt ions of plasma were removed using a multi-stage membrane separation experimental machine (DMJ60-2, Jinan Bona Biological Technology Co., Ltd, Shandong, China), and freeze-dried to prepare the plasma powder with the lowest ash content. The cut-off molecular weight of the coil ultrafiltration membrane components was 10,000 Da, and the selected molecular weight of the coil nanofiltration membrane was 150 Da. The membrane’s working pressure was 0.6 MPa and the pH of the plasma was 9. The ash content of the desalted and the un-desalted freeze-dried plasma protein powder were determined according to the Chinese standard GB 5009.4 [20]. Different proportions of the freeze-dried plasma protein powder, without the treatment of ultrafiltration, were mixed with the freeze-dried desalted plasma powder to prepare the plasma powder with different ash contents (calculated values: 7%, 9%, 12%, 15%, and 18%, respectively; measured values with the same unit protein content: 6.17%, 8.92%, 11.92%, 15.24%, and 18.82%, respectively).

### 2.3. Water-Holding Capacity and Texture Analyses

Plasma protein powders were dissolved in ultrapure water. The protein concentrations of liquid plasma and plasma powder solutions were adjusted to 60 mg/mL, and the pH of these solutions was adjusted to 9. Samples were heated at 80 °C for 45 min to form gels. The gels were immediately cooled to room temperature and stored in the refrigerator at 4 °C overnight to age for further analysis [16]. The water holding capacity (WHC) of the gels was calculated via a centrifugal method [21]. The pieces of the gel after weighing were centrifuged at 1000× *g* for 10 min at 4 °C. WHC was calculated as the percentage of water retained based on the water content in the gels prior to centrifugation. Three replicates were measured for each sample.

Textures (hardness and elasticity) were analyzed by the texture profile analysis (TPA) test using a texture analyzer (TA-XT2i/5, Stable Micro Systems, Godalming, UK) with a cylindrical probe (P/0.5R) according to the method of Li et al [22]. The parameters were as follows: pre-test speed, 1.0 mm/s; test speed, 0.5 mm/s; withdrawal speed, 1.0 mm/s; depth of probe penetration, 5 mm; minimum trigger force, 5 g; and data acquisition rate, 200 points/s. For each gel, the texture was measured in triplicate.

### 2.4. Microstructure

The microstructures of the gels were investigated by scanning electron microscopy (SEM). The gels formed from liquid and dehydrated plasma were fixed in 3% glutaraldehyde for 48 h at 4 °C, re-fixed in osmic acid for 2 h, and washed three times with phosphate-buffered saline. Then, they were dehydrated using an ethanol series (50%, 70%, 80%, 90%, and 100% (v/v ethanol, successively)). Freeze-drying and sputter-coating were performed according to the procedure of Han et al [23]. Samples were dried using carbon dioxide critical point drying, and coated with Au in a vacuum ion sputtering system. These specimens were observed in a Hitachi SU8010 SEM (Hitachi Ltd., Tokyo, Japan), operating at a voltage of 15 kV [24].

### 2.5. Fourier Transform Infrared Measurements

The gels of plasma powders and liquid plasma were prepared in an 80 °C water bath for 45 min and were measured using a Fourier transform infrared spectrometer detector (Tensor 27, Bruker, Germany). The resolution was 4 cm^−1^, the scanning range was 4000–600 cm^−1^, and the signal was cumulatively scanned 64 times. Spectral OPUS 7.0 software was used for background subtraction and CO_2_ atmosphere compensation. Peakfit 4.2 was used for baseline correction, a second derivative peak fitting of gauss points of the amide І band (1600–1700 cm^−1^), and the estimated position and number of the stack peak of the amide І band.

### 2.6. Differential Scanning Calorimetry

The level of denaturation for plasma proteins was studied using differential scanning calorimetry (DSC). One hundred microliters of liquid and dehydrated plasma solutions with the same protein concentration (60 mg/mL) were placed in DSC pans, hermetically sealed, and subsequently analyzed using a DSC Q200 (TA Instrument, New Castle, DE, USA). A pan that contained 100 μL of distilled water was used as a control. All pans were heated from 25 to 105 °C at 3 °C/min. The thermal denaturation point (T_d_, °C), which was the minimal heat flows in the DSC thermogram, and the enthalpy of denaturation (∆H, J/g) determined by the integration of the area belonging to the changes in heat flow, as a function of the temperature, was calculated from the thermogram [25].

### 2.7. Statistical Analysis

All heat-induced gels were carried out in triplicate and each sample was measured in triplicate. Analysis of variance (ANOVA) was performed using SPSS 22 for Windows (SPSS Inc., Chicago, IL, USA). The differences of means were evaluated by the Duncan test (*p* ≤ 0.05).

## 3. Results and Discussion

### 3.1. Effect of Drying Methods on Heat-Induced Gelation of Plasma Proteins

Previous studies reported that the gel properties of plasma protein can be finely adjusted by pH [10,11,16]. The hardness of heat-induced gels can be increased by increasing the pH levels [8]. In the present study, pH 9 was selected, as the gels have good gel properties in this condition.

**Figure 1:**
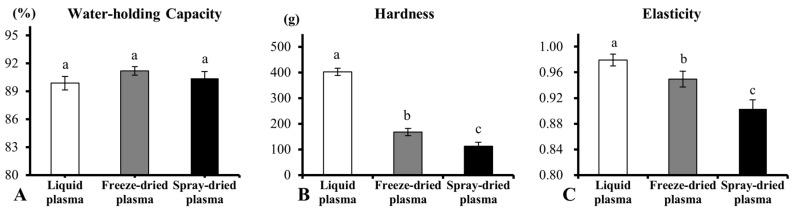
The effect of drying methods on water-holding capacity (WHC) and texture of heat-induced porcine plasma protein gels. (**A**) WHC of the gels, (**B**) Hardness of the gels, (**C**) Elasticity of the gels. Different letters indicate significant differences (*p* < 0.05).

WHC is one of the most important functional properties of heat-induced protein gels [26]. In the present study, the WHC was not significantly different among the three groups (*p* > 0.05) (Figure 1A). The result was in agreement with Parés et al. [16], who reported that the WHC was not different between gels obtained from liquid plasma and spray-dried plasma, at any given pH (4.5, 5.5, 6, and 7.4). However, the result was not in agreement with Gong et al. [27] working with peanut protein isolate, which showed that the WHC of freeze-dried peanut protein isolate were significantly higher than those of the spray-dried one. The high temperature of spray-drying can affect the structure and properties of the protein [28,29]. This could be due to the differences in the protein and drying parameters.

The hardness and elasticity of heat-induced gels significantly decreased in the freeze-dried and the spray-dried plasma powders (*p* < 0.05) (Figure 1B,C). Besides this, the hardness and elasticity of the gels from the spray-dried plasma were less than that of the freeze-dried plasma. Due to the ion concentration changes, the degeneration of plasma protein occurs during dehydration of the liquid plasma [16]. The spray-drying may cause more plasma proteins to be denatured on heating compared to freeze-drying [29,30]. The denatured proteins effect the aggregation of proteins [16,30], which may lead to a reduction in gel hardness and elasticity.

**Figure 2:**
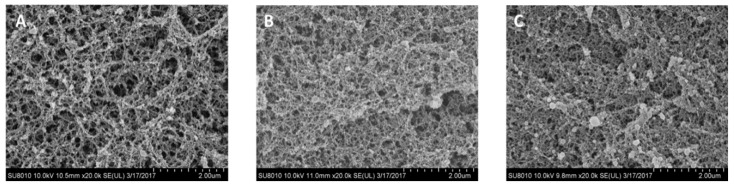
Scanning electron micrograph (magnification, ×20,000) of heat-induced porcine plasma protein gels from different drying methods. (**A**) Gels from liquid plasma, (**B**) Gels from freeze-dried plasma, (**C**) Gels from spray-dried plasma.

The microstructures of the heat-induced liquid plasma and dehydrated plasma powder gels are shown in Figure 2. The gels exhibit a clearly ordered porous structure, and slight differences were observed among different samples. The pores of gels from liquid plasma were slightly larger than those of dehydrated plasma powders (Figure 2B,C). Wang et al. [11] reported that fine-stranded gels were formed when the pH was higher than 6.0, but a disordered and particulate gel network with several large pores was formed at a low pH, i.e., 5.5. The present result showed that fine-stranded gels were formed for liquid plasma at pH 9, which is consistent with the previous study. Parés et al. [16] showed that no notable differences in the microscopic structure of gels from liquid and spray-dried plasma were observed. In the present study, there were only slight differences between the treatments. The microstructure of the freeze-dried plasma gel was more compact than that of the liquid plasma gel. These structural modifications could explain no differences in WHC, but the hardness and elasticity of dehydrated plasma are lower than those of liquid plasma.

**Figure 3:**
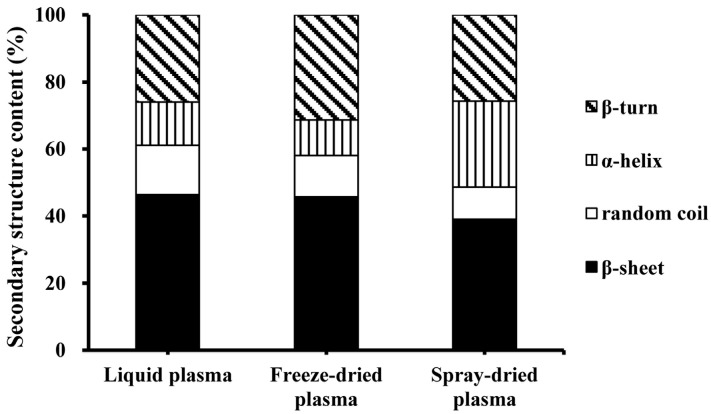
The effect of drying methods on the secondary structure of heat-induced porcine plasma protein gels.

The secondary structure of the gels from liquid plasma and spray-dried and freeze-dried plasma powders are shown in Figure 3. The secondary structure of gels from the liquid plasma and freeze-dried plasma powders were similar. The main secondary structure was β-sheet, followed by β-turn; there were fewer random coils and α-helixes. The secondary structure of the gels from spray-dried plasma protein powders exhibited a different composition. The main secondary structure was β-sheet, followed by β-turn and α-helix, and there were fewer random coils. A previous study has shown that the spray-dried peanut protein isolate had a relatively more unfolded or flexible structure than the freeze-dried peanut protein isolate [27]. In the present study, the result also showed that spray-drying affected the structure of protein gels. The reason is that the thermal denaturation process significantly affected the protein’s secondary structure [31].

**Figure 4:**
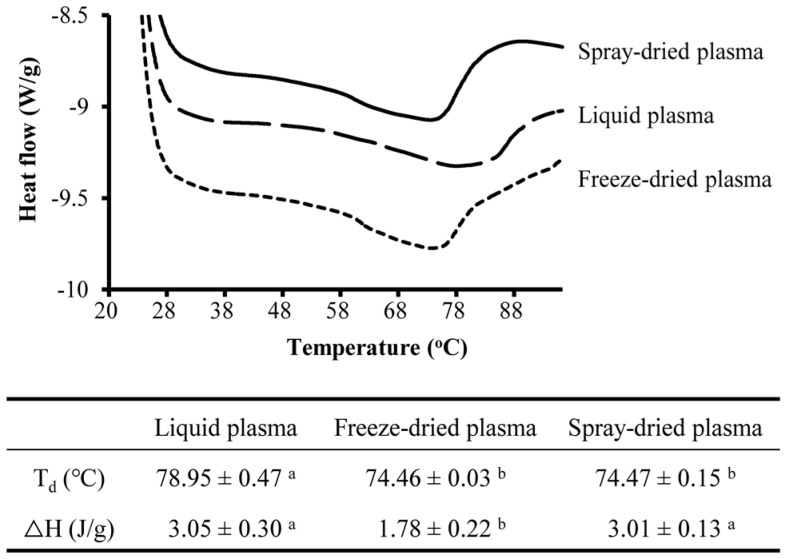
The effect of drying methods on the thermal denaturating temperature of plasma protein. Different letters indicate significant differences (*p* < 0.05).

The DSC curves of the liquid plasma and spray-dried and freeze-dried plasma powders are shown in Figure 4. The T_d_ of liquid plasma was significantly higher than that of the dehydrated plasma (*p* < 0.05), indicating that the thermal stability of the liquid plasma was better. The thermal denaturation of protein is closely related to the change in its spatial conformation. The thermal stability of plasma proteins was changed during drying, which led to the different thermal denaturation states. The ∆H of the liquid plasma and spray-dried plasma were higher than that of the freeze-dried plasma. These results were not in agreement with the study of Parés et al. [16], who reported that the differences of the peak temperature and enthalpy calculated for the liquid plasma and spray-dried plasma were not significant at the same pH (4.5, 5.5, 6.0, and 7.4).

### 3.2. Effect of Ash Contents on Heat-Induced Gelation of Plasma Protein

**Figure 5:**
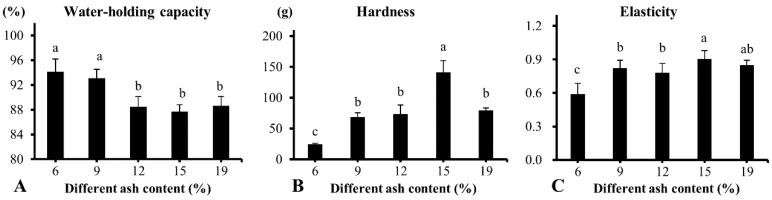
Effect of different ash contents on the water-holding capacity (WHC) and texture of heat-induced porcine plasma protein gels. (**A**) WHC of the gels, (**B**) Hardness of the gels, (**C**) Elasticity of the gels. Different letters indicate significant differences (*p* < 0.05). The corresponding unit ash contents of 6%, 9%, 12%, 15%, and 19% were 6.17%, 8.92%, 11.92%, 8.92%, and 18.82%, respectively, while adjusting the concentration of each protein to be consistent.

In the current study, the freeze-dried plasma powder showed better gel properties (higher hardness and elasticity) than the spray-dried plasma powder. On this basis, the effect of the ash content of freeze-dried plasma on the gelation properties was studied. The content of the ash is an important indicator of plasma protein powder products. The main component of ash is sodium, then potassium, and calcium. Research shows that sodium chloride affects the gel properties of the protein [32,33]. In Figure 5A, the WHC of heat-induced gels decreased with the increasing ash contents of freeze-dried plasma protein powders. The WHC for the samples with 6% and 9% ash content was significantly higher than that for the samples with 12%, 15%, and 19% ash content (*p* < 0.05). No significant difference was found as ash content increased from 12% to 19%. In the present study, we found that the gel from the 6% plasma protein powder had a soft texture and high viscidity (Figure 5B,C). The ash content of plasma powder significantly influenced the hardness and elasticity of heat-induced gels (*p* < 0.05). The hardness of heat-induced gels increased first and then decreased with the increasing ash content. The gel of the sample with 6% ash content has the lowest hardness and elasticity. The gel of the sample with 15% ash content has the highest hardness and elasticity. The elasticity values were not different between 9%, 12%, and 15% ash content samples. Those results indicated that the texture of gels with low ash content were worse compared to high ash content plasma protein powder. However, the gels with low ash content had a good WHC. This result could be in agreement with that obtained by Meng et al. [34], emphasizing that the WHC decreased with the ion concentration, increasing if the ion concentration was larger than 0.3 mol/L.

During the formation of the gel, the sodium neutralizes the charge on the surface of the protein, leading to the attraction between protein molecules enhancing, and the molecules rapidly aggregating to form a hard gel. When the ash content increases to a certain extent, it is difficult to form gel because of the high concentration of salt-stabilized protein molecular conformations. A previous study showed that high concentrations of NaCl decreased the water-holding capacity of egg-white gels [35], and the present study showed the same result. The reason for this can be attributed to the unstable water molecules trapped in large cavities in the protein gel network [36], and the high solid content existing in the plasma proteins.

**Figure 6:**
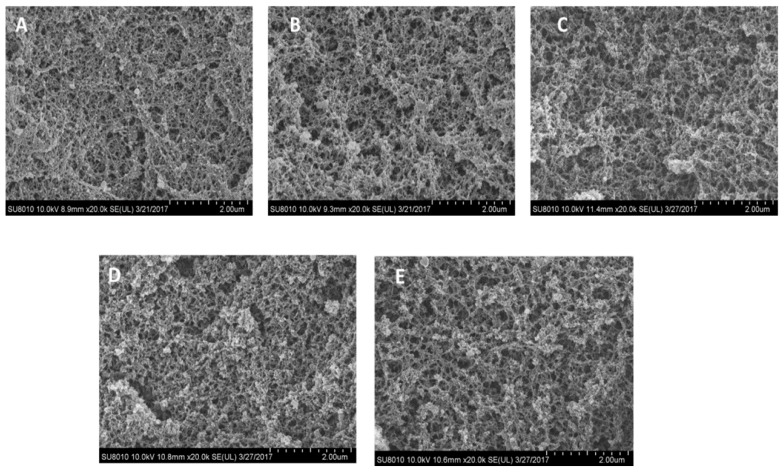
Scanning electron micrographs (magnification, ×20,000) of heat-induced porcine plasma protein gels with different ash contents. (**A**) Gels from plasma powder with 6% ash content, (**B**) Gels from plasma powder with 6% ash content, (**C**) Gels from plasma powder with 12% ash content, (**D**) Gels from plasma powder with 15% ash content, (**E**) Gels from plasma powder with 19% ash content. The ash of A–E were 6%, 9%, 12%, 15%, and 19%, and their corresponding unit ash content was 6.17%, 8.92%, 11.92%, 15.24%, and 18.82%, respectively, while adjusting the concentration of each protein to be consistent.

The microstructures of the heat-induced gels for different ash content plasma protein powders are shown in Figure 6. The results showed that ordered and three-dimensional network gels were formed. The micrographs did not distinguish between different ash content plasma powders. Therefore, if we just focus on the microstructure of plasma gels, low ash content had little effect on the gels’ microstructure.

**Figure 7:**
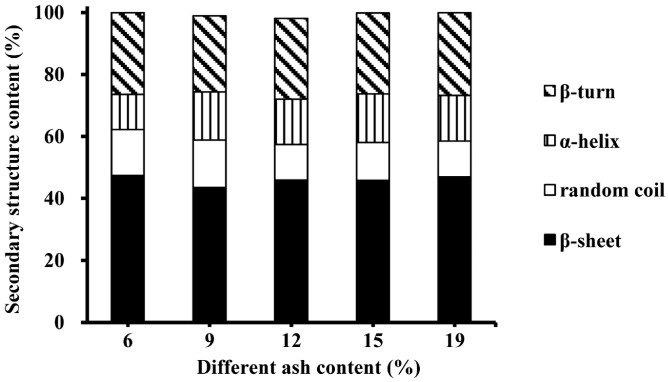
Effect of different ash contents on the secondary structure of heat-induced porcine plasma protein gels.

The secondary structures of plasma protein powders with different ash contents are shown in Figure 7. The secondary structures of gels from different ash content plasma protein powders were similar. Furthermore, the main secondary structure of the heat-induced gel was β-sheet, followed by β-turn, and there were fewer random coils and α-helixes. A previous study reported that some physical and chemical conditions–pH, ion concentration, sugar content, and metal content of protein solution–affected the protein’s secondary structure [37]. However, the present result showed that the influence of the ash content (6–19%) on the composition and content of the secondary structure was insignificant.

## 4. Conclusions

The gels from the dehydrated plasma powders exhibited lower hardness and elasticity than that from the liquid plasma. A possible cause is that dehydrated plasma had lower thermostability and formed a gel with dense microstructures. The gel from the spray-dried plasma powder exhibited lower hardness and elasticity than that from the freeze-dried plasma powder. The secondary structure of the gels from the spray-dried plasma protein powders exhibited more α-helixes and less β-turns than that from the freeze-dried plasma protein and liquid plasma. Compared with the gels of high ash content plasma protein powders, the gel from the 6% ash content plasma powder had the highest water-holding capacity and had the lowest hardness and elasticity. However, the secondary structure and microstructures of the heat-induced gels were not affected by the ash contents of plasma powders. Therefore, drying methods and the ash contents of plasma protein powders affect the quality of heat-induced gel properties. Further studies on food model systems are necessary to confirm the results obtained in the present study.
